# Cross-level research on the impact of self-serving leadership on employee innovation behavior: The roles of workplace anxiety and team psychological safety

**DOI:** 10.3389/fpsyg.2022.1069022

**Published:** 2023-01-12

**Authors:** Liangcan Liu, Zhitao Wan, Li Wang

**Affiliations:** ^1^School of Business Administration, Guizhou University of Finance and Economics, Guiyang, China; ^2^School of Economics and Finance, Guizhou University of Commerce, Guiyang, China

**Keywords:** self-serving leadership, team psychological safety, workplace anxiety, employee innovation behavior, cross-level

## Abstract

Employee innovative behavior is significant in maintaining an organization's sustainable development. This study explored the impact of team psychological safety and workplace anxiety on the association between self-serving leadership and employee innovation behavior by synthesizing social information processing theory, conservation of resources theory, and ego depletion theory. We conducted a hierarchical linear model analysis using three-wave paired data collected from 86 leaders and 392 employees. The research results showed that self-serving leadership is negatively correlated with employee innovation behavior. Meanwhile, team psychological safety and workplace anxiety mediated this relationship. In addition, team psychological safety mitigates the impact of workplace anxiety on employee innovation behavior and the indirect impact of self-serving leadership on employee innovation behavior *via* workplace anxiety. These findings have a number of theoretical and practical implications in the domains of self-serving leadership and employee innovation behavior.

## 1. Introduction

Innovation is crucial to the survival and prosperity of an organization (Hjalager, 2010). As the direct implementer of innovation activities, employees' innovation behavior determines the innovation level of an enterprise (Shalley et al., 2004). Therefore, managers and researchers have begun to pay closer attention to employees' innovative behavior. As an important situational factor in the organization, one of the important functions of leadership is to promote innovative behaviors in employees and obtain sustainable organizational competitive advantages (Zhang X., 2010). Leadership is an important predictor of employee innovation behavior (Liden et al., 2014). A large number of studies focus on the link between positive leadership and employee innovative behavior, such as transformational leadership (Pieterse et al., 2009), empowering leadership (Zhang X., 2010), ethical leadership (Yidong and Xinxin, 2013), and inclusive leadership (Carmeli et al., 2010). However, leadership has positive and negative effects, with negative leadership impacting employees' behavior more than positive leadership (Jiang and Gu, 2016). Researchers found that leaders do not always benefit the organization (Rafferty and Restubog, 2011) and sometimes utilize organizational resources to seek their own interests (Camps et al., 2012). As a result, self-serving leadership began to attract the attention of scholars.

As a prevalent form of leadership in organizations (Decoster et al., 2021), self-serving leadership refers to leaders who put their interests above the needs of their subordinates and organizational benefit (Camps et al., 2012). As an emerging field of leadership research, there is a growing body of research on self-serving leadership, and the impact of self-serving leadership on organizations requires researchers to pay more attention. Existing research reported that self-serving leadership has a series of detrimental effects on employees and teams (Schyns and Schilling, 2013), such as causing psychological harm and negative moods in subordinates (Camps et al., 2012), inhibiting employees' willingness to cooperate (Decoster et al., 2014), reducing employees' contentment with supervisors and organizational citizenship behavior toward leaders (Ritzenhöfer et al., 2019), motivating subordinates' tendency to quit (Ritzenhöfer et al., 2019), showing counterproductive work behavior (Mao et al., 2019b), triggering deviant behaviors (Zhou et al., 2021), and also weakening team creativity (Peng et al., 2019). However, whether self-serving leadership impacts employees' innovative behavior needs to be proven. As a typical form of destructive leadership (Schmid et al., 2019), self-serving leadership can trigger negative emotions and uncertainty in employees (Camps et al., 2012), making them feel insecure and thus inhibiting their innovative behavior. In addition, employees are nested within a work team (Zhang Z.-X., 2010), and their innovative behavior can be influenced by a high-level construct (i.e., self-serving leadership). Since self-serving leadership can affect individual employees and the team, we infer that self-serving leadership should be a multilevel variable.

Meanwhile, according to leadership theory, leadership can influence employees' innovative behavior through both individual and team factors (Xu et al., 2020). Unfortunately, existing studies mainly focus on self-serving leadership at the individual level, ignoring the impact of team-level characteristics of self-serving leadership, and have not clarified how the high-level construct of self-serving leadership influences the innovation behavior of subordinates, including at the individual level (e.g., attitude, cognition, and emotion) and team level (e.g., psychological safety atmosphere). Therefore, this study will be helpful in systematically exploring the cross-level impact of self-serving leadership on employee innovation behavior.

According to the theory of social information processing, social cues from leaders affect employees' interpretations of the work environment, resulting in their perception and understanding and then affecting their subsequent behaviors (Salancik and Pfeffer, 1978). Team employees depend on the information gathered by team leaders to form perceptions of the team environment and adjust them accordingly (Gu et al., 2016). Self-serving leadership is detrimental to employees' wellbeing (Mao et al., 2017), making them feel that the organization cannot protect their interests and instilling a profound fear in them (Peng et al., 2019). In addition, self-serving leaders usually ignore the wellbeing of employees (Camps et al., 2012). They do not recognize employees' efforts, resulting in the decline of common psychological safety (Peng et al., 2019). However, the sense of team psychological safety can effectively help employees coordinate interpersonal relationships, and members can freely express their opinions and ideas without worrying about a negative impact on their work status or reputation (Roussin and Webber, 2012). Therefore, members can openly discuss and exchange information related to tasks, promoting their cooperation and learning (Roussin et al., 2014) while daring to express their opinions (Patterson et al., 2004) to stimulate members' innovative behavior (Carmeli et al., 2010).

This study brought team psychological safety into our study framework and explored the association between self-serving leadership and employee innovation behavior, as well as examined its behavioral effects on the relationship between the two. As the controller and distributor of resources, leaders' selfish behaviors damage the interests of subordinates, and trigger anxiety among employees (Mao et al., 2019a), thus inhibiting employee innovation behavior (Samma et al., 2020). Based on this notion, the present study also explored the mediation effects of workplace anxiety on self-serving leadership and followers' innovation behavior.

In addition, we believe that team psychological safety is a significant moderator of workplace anxiety and employee innovation behavior. According to self-depletion theory, team psychological safety, as a work resource at the team level (Halbesleben et al., 2014), reduces members' interpersonal risk relating to their expressions of anxiety—team members do not worry that expressing their concern will lead to a denial of their ability or degradation of their image by team leaders and colleagues. As a result, employees save their limited self-control resources and have more resources to invest in follow-up work, thus stimulating more innovative behaviors at work (Amabile, 1993). By studying the interaction of team psychological safety and employees' anxiety in the workplace context, this study provides a new perspective for organizations on alleviating the detrimental effect of workplace anxiety on employee innovation behavior.

Our research integrated social information processing theory, resource conservation theory, and ego depletion theory to investigate the effect of egoistic leaders on employees' innovative behavior. This study's innovation lies in studying the impact of self-serving leadership on employee innovation behavior and discussing the mediating mechanism and boundary conditions of the relationship, which enriches the theoretical research results of self-serving leadership. The questions explored in the research are as follows. Q1: How does self-serving leadership impact employee innovation behavior? Q2: What is the mediating mechanism in the impact of self-serving leadership on employees' innovative behavior? Q3: What are the boundaries for the relationship between self-serving leadership and employee innovation behavior?

To answer these questions, SPSS 23.0, AMOS 24.0, HLM 6.08, and R 3.6.3 were adopted to carry out statistical analysis on the collected questionnaires. First, the reliability of model variables was analyzed using Cronbach's α. Second, the validity of model variables was analyzed using CFA. Then, the common method bias of the variables was tested. Third, the basic statistical information and correlation relationships of variables were judged using descriptive statistics and correlation analysis. Finally, a hierarchical linear model (HLM) was used to test the hypothesis. Additionally, the Monte Carlo method was adopted to test the effects of mediating and moderating.

There are three reasons why the hierarchical linear model (HLM) was adopted in this study. First, from the theoretical perspective, the important influence of team leaders as “atmosphere engineers” on employee behavior is discussed (Kozlowski and Doherty, 1989). Studies showed that self-serving leadership can exist at the team level (Peng et al., 2019). Second, in terms of data collection, we collected the questionnaire data through cluster sampling. Individuals are nested within work groups. In other words, the data were nested. Therefore, the model was designed as a hierarchical linear one (Bryk and Raudenbush, 1992); this method has also been adopted in other studies (for a similar approach, see Table 1).

**Table 1:** Relevant previous studies adopted HLM analysis.

**References**	**Team level**	**Individual level**
Hsiung (2012)	Authentic leadership; Procedural justice climate	Positive mood; Leader–member exchange; Employee Voice behavior
Li et al. (2017)	Differentiated empowering leadership	Trust in leaders; Chinese traditionality; In-role performance; Extra-role performance; Counterproductive work behaviors
Tourigny et al. (2019)	Ethical leadership; Corporate social responsibility	Organizational trust; Taking responsibility; Organizational citizenship behavior
Zhang and Song (2020)	Humble leadership; Error management climate	Psychological safety; Work wellbeing
Liu et al. (2022)	Humble leader behavior; Team cognitive diversity; Team potency; Team performance	Organization-based self-esteem; Individual performance
Meng et al. (2022)	Transformational leadership	Meaningfulness in work; meaningfulness at work; work engagement

Source: The authors.

This study is structured as follows: In Section 2, we review relevant literature and hypotheses. Section 3 presents the methodology. The results are reported in Section 4. The discussion and theoretical and managerial contributions, as well as limitations of the present study and future research on self-serving leadership, are presented in Section 5.

## 2. Literature review and hypotheses development

### 2.1. Self-serving leadership and employee innovation behavior

Self-serving leaders put their own interests above those of their followers and the organization for which they work (Camps et al., 2012), which adversely affects employees' wellbeing, causes harmful and long-term consequences for the organization (Haynes et al., 2015), and has many adverse effects on employees (Haynes et al., 2015). However, employee innovation behavior is referred to as the process in which employees inspire novel and valuable ideas in workplace contexts and attempt to put them into practice (Shi, 2012), including the generation, promotion, and realization of innovative thinking (De Vries et al., 2016). Since employee innovation behavior exceeds the prescribed role expectations, it belongs to out-of-role behavior (Wang and Chang, 2017). Existing research showed that individual factors, such as personality (Raja and Johns, 2010; Saura et al., 2021), self-efficacy (Wang et al., 2014), perceptions of differences in order atmosphere (Ma and Su, 2020), employees' positive perceptions of their companies' support (Saura et al., 2022), and emotions (George and Zhou, 2007), as well as organizational situational factors, such as innovation climate (Baer and Frese, 2003) and leadership, influence employees' innovative behavior. However, as an important component of an organization, leadership is a key factor in stimulating employees' innovative behavior (Choi et al., 2015; Shen et al., 2019).

According to social information processing theory (Salancik and Pfeffer, 1978), the surrounding social environment largely influences people's attitudes and behaviors: people decide what kind of attitude and behavior they have to adopt by processing and interpreting specific social information. For example, leaders are the primary source of information for employees (Jiang and Gu, 2016). Thus, team members interpret the information that self-serving leaders provide and adjust their perceptions and behaviors accordingly. Therefore, we expect that team leaders exhibiting self-serving behaviors will harm team members' innovative behaviors. The main reasons are as follows.

First, when team leaders are self-serving, team members fear that the leader will steal their benefits (Mao et al., 2019a), putting the team members' work performance at risk of not being recognized by the leader (Mao et al., 2017), causing members to exhibit negative emotions and experience a sense of uncertainty (Camps et al., 2012). Uncertainty affects team members' cognition, emotions, and behaviors. It also reduces team members' sense of control and predictability in the environment, causes them to lose their sense of security, and triggers a sense of uncertainty (Hogg, 2007), leading to stress and work distractions (Mao et al., 2019a), which ultimately detaches employees from work and makes them unwilling to innovate (May et al., 2004). Meanwhile, the expectations of innovation are uncertain, implying high risks and the possibility of failure (Carmeli et al., 2010). In the absence of a sense of security, employees become less willing to take responsibility and increase their risk-averse behavior (Mao et al., 2019a), leading to a decline in employee innovation behavior.

Second, self-serving leaders who prioritize their own interests above the organization's interests and others make employees vulnerable to the infringement of their interests (Mao et al., 2017). As a result, team members trust their leaders less (Decoster et al., 2021) and produce fewer positive work outcomes (Lau and Liden, 2008), such as cooperative behaviors (Coleman, 1990) and organizational citizenship behaviors (McAllister, 1995). In addition, it prompts employees to take actions to restore the imbalance between their efforts and expected returns (Carlsmith et al., 2002) and reduce the willingness of members to provide services for the organization (Haynes et al., 2015), such as a willingness to cooperate and extra-role behaviors that are beneficial to the organization but not within the organization's formal salary assessment (Decoster et al., 2014), such as innovative behavior. In addition, when faced with a self-serving leader, the members may think that they are not valued (Camps et al., 2012), which leads to the perception that their work is unimportant and worthless to the organization. As a result, they will pay less attention to their work and thus reduce their innovative behaviors.

Based on social learning theory (Bandura, 1977), employees observe the leader's behavior and learn from it. Thus, employees will learn self-serving behaviors by imitating a self-serving supervisor, forming self-serving values, and then guiding their behaviors (Haynes et al., 2015). Hence, self-serving leadership promotes an unhealthy organizational climate in which each member's interests above others are acceptable, causing team members to adopt a self-serving code of conduct (Vardaman et al., 2014). Under these self-serving values, there will be less knowledge exchange (Peng et al., 2019) since employees tend to hide knowledge to avoid personal loss. This reduced sharing increases the cost of knowledge acquisition for employees and hinders the free flow of knowledge (Zhao, 2020). Since this behavior is not conducive to employees acquiring new knowledge, it reduces employee innovation behavior. In sum, we hypothesize the following:

H_1_: Self-serving leadership is negatively related to employee innovation behavior.

### 2.2. The cross-level mediating effect of team psychological safety

Team psychological safety is defined as members' common perceptions that taking an interpersonal risk in a team environment is safe. They believe they can express what they think and feel and that the team would not refuse, embarrass, or punish anyone who dares to state their opinions; the basis of this belief is trust and mutual respect between members (Edmondson, 1999). Leadership behavior is the key premise of psychological safety (Ortega et al., 2014). For example, Edmondson (2003) believes that the behaviors of team leaders can trigger team members' awareness of interpersonal risks, thus affecting their psychological safety. Meanwhile, leaders' different attitudes toward tasks and members have distinct influences on shaping team atmosphere and psychological states (Qing et al., 2012). Therefore, when team leaders shape the image of openness and fallibility, they can effectively promote a psychologically safe atmosphere for the team (Edmondson and Roloff, 2009). Meanwhile, leaders who prioritize the interests of their team members create an atmosphere of psychological safety within the group (Hu et al., 2018). On the contrary, when leaders lack sympathy or exploit members, it causes psychological insecurity among members (Jiang and Gu, 2016).

Social information processing theory posits that leadership behavior is an important information source that affects team members' behavior in the work environment. How team members interpret information helps them understand their work environment and shapes their behavior (Salancik and Pfeffer, 1978). In other words, team members use cues from their leaders to test their interpretations of the team environment and adjust their perceptions (Gu et al., 2016). Specifically, self-serving leaders will occupy organizational resources (Rus et al., 2010), sacrifice others' interests to achieve their goals, shift blame, and use deceptive means to satisfy their interests (Schilling, 2009). As a result, their interests threaten team members (Mao et al., 2017). Through the interpretation of this information, team members believe that leaders do not recognize their contributions, making them feel as if the leaders are taking advantage of them and inducing fear of exposing mistakes within the team. Therefore, employees tend to cover up their errors or even blame each other, which leads to the alienation of interpersonal relationships among employees (Du et al., 2015). It makes them realize that the team environment cannot bear the risks of interpersonal communication, which leads to a shared psychological insecurity among team members (Peng et al., 2019).

Social information affects individual behavior everywhere. Innovation is a risky activity with unpredictable outcomes (George, 2007). Employees can be encouraged to put forward new ideas or viewpoints when the working environment tolerates the risk of undertaking innovative activities (West, 1990). In addition, employees can improve their innovative skills (Edmondson, 1999), increase work input, and fully engage in originality through active collaboration and creative problem-solving (Brown and Leigh, 1996). Psychologically safe work environments include trust and encourage employees to take risks without fear of adverse effects on their work status or workplace reputation (Roussin and Webber, 2012). In this work environment, team members do not have to worry about being criticized, blamed, or punished by other members for presenting a different point of view; they tend to express their true thoughts, ask questions, and frankly discuss mistakes in their work. In addition, team members can seek help and feedback from other members (Ortega et al., 2014), enabling the free exchange of task-related information and thus promoting cooperation and learning among members (Roussin et al., 2014).

Previous studies showed that team psychological safety can promote exploratory and exploitive innovation (Nemanich and Vera, 2009). The higher the team's psychological safety, the more innovative their behaviors are (Vinarski-Peretz and Carmeli, 2011). Based on the above analysis, self-serving leadership inhibits employee innovation *via* team psychological safety. In sum, we hypothesize the following:

H_2_: Team psychological safety cross-level mediates the relationship between self-serving leadership and employee innovation behavior.

### 2.3. The cross-level mediating effect of workplace anxiety

Workplace anxiety includes feelings of tension and apprehension about achieving job tasks (Muschalla and Linden, 2012). There are two types of anxiety: trait and state. This study considers workplace anxiety as state anxiety in an organizational context, an unabiding emotional status. It includes cognitive anxiety and physiological arousal (Endler and Kocovski, 2001) and reflects general feelings of work-related anxiety (Spielberger, 1972), often occurring when employees feel threatened and experience stress at work (Cheng and McCarthy, 2018).

Based on the conservation of resources theory, people tend to strive to maintain, protect, and obtain resources that contribute to realizing their personal goals. Therefore, individuals with more resources are more likely to obtain the preservation and appreciation of those resources and are less affected by the loss of those resources. On the contrary, individuals with fewer resources are more vulnerable to the harmful effects of actual or potential losses (Hobfoll, 1989). As a competitor for the resources available to employees (Mao et al., 2019a), self-serving leadership may induce employees' workplace anxiety and impede their innovative behavior. Self-serving leaders put their benefits above the interests of the organization and others, affecting subordinates' perceptions of available resources (Mao et al., 2019a), threatening subordinates' feelings of interest deprivation, and thus triggering subordinates' stress responses (Hobfoll, 1989). Therefore, self-serving leaders impact the psychological status of employees (Brotheridge and Lee, 2002). If employees believe that the final work result is less than their expectations, they tend to adopt relatively negative resource processing motivation, which is manifested explicitly as negative emotions and increased workplace anxiety (Ye et al., 2021); this acts as a self-help signal when their survival is threatened (Cheng and McCarthy, 2018).

Workplace anxiety is detrimental to employee innovation behavior. First, innovation needs to change the routine and the status quo, which will affect the interests of some employees. It requires employees to be willing to take risks, know-how to communicate and cooperate with others, and invest numerous resources (Agarwal, 2016). According to the conservation of resources theory, anxiety consumes more cognitive resources than any negative emotion (Ferris et al., 2008). Therefore, it reduces employees' investment of cognitive resources in innovation and negatively affects their innovation behavior.

Second, according to attention control theory, when there is a threat-related stimulus, anxiety will significantly reduce the individual's attention to control—anxious members will allocate their limited attention to the source of the threat (Eysenck et al., 2007). Therefore, when leaders threaten employees' interests and cause intense anxiety, they will pay more attention to how to weaken the leaders' threat to their interests, thus reducing their attention to work and innovative behaviors. Finally, compared with calm employees, anxious employees think less efficiently, thus impeding their innovative behaviors (Eysenck et al., 2007). In conclusion, leaders' self-serving behaviors increase employees' workplace anxiety, while employees' workplace anxiety inhibits their innovative behaviors. In sum, we hypothesize the following:

H_3_: Workplace anxiety is cross-level and mediates the relationship between self-serving leadership and employee innovation behavior.

### 2.4. The cross-level moderating effect of team psychological safety

Based on the theory of self-depletion, the ability of employees to exercise self-control is a depletable resource (Baumeister et al., 1998), and individual self-control consumes specific control resources (Baumeister et al., 2007). Ego depletion of control resources after employees perform self-control tasks (Muraven and Baumeister, 2000) depletes available resources in another area (Ren et al., 2014). As a result, the performance of subsequent self-control tasks worsens (Baumeister et al., 2007), resulting in many adverse consequences (Klotz et al., 2018). In addition, team psychological safety is a kind of social support and work resource at the team level (Halbesleben et al., 2014); it is an opportunity perceived by employees or an actual supportive behavior (Hobfoll, 2002).

Although the literature on psychological safety mainly focuses on employees' perceptions of taking interpersonal risks, such as asking questions or making mistakes (Edmondson, 1999), we can also consider emotional expression in this context (Grandey et al., 2012). A team with better psychological safety has mutual respect and trust among its members. Employees perceive trust and support from colleagues and have a positive relationship with them (Banks et al., 2014). They believe that expressing their emotions will not cause difficulties or embarrassment for other members (Edmondson, 1999). They do not worry about voicing their anxiety, even if team leaders and colleagues might think less of them. They even believe bold expressions to be beneficial, which effectively reduces the psychological pressure of expressing their anxiety and makes their psychological state more stable, thus reducing the impact of negative emotions (Wei et al., 2019).

In a team that is better at psychological safety, individuals are less concerned about the interpersonal risks associated with expressing their anxiety, thus saving their limited self-control resources. At the same time, as an organizational support resource, team psychological safety gives employees a chance to replenish psychological resources (Througakos et al., 2008) so that team members can compensate for resource loss caused by anxiety (Grandey et al., 2012). This enables employees to have more resources to invest in follow-up work, which is conducive to employee involvement (Lin and Johnson, 2015). In addition, employees actively coordinate various resources to meet work challenges and inspire more innovation at work (Amabile, 1993).

In addition, as a negative emotion, anxiety consumes employees' cognitive/emotional resources (Weiss and Cropanzano, 1996), reducing their cognitive/emotional input into innovation. Furthermore, when people feel uninhibited (Grandey et al., 2012), they experience a reduced loss of self-control resources, effectively improving their subsequent work performance (Througakos et al., 2008). However, in an environment with low team psychological safety, team members will have a higher interpersonal risk perception. As a result, they may suppress their emotions, resulting in more consumption of self-control resources and poor performance in subsequent tasks (Goldberg and Grandey, 2007). Negative emotions (including workplace anxiety) can reduce the quality of interpersonal relationships between team members (Tse and Dasborough, 2008), leading employees to believe that freely expressing their concerns may bear uncertain interpersonal risks.

As a result, employees who suffer from workplace anxiety will suppress their emotions to avoid unnecessary interpersonal issues in the team. However, the inhibited expression will consume self-control resources, leading to employees' lack of self-control in follow-up tasks. This action will harm employee work engagement (Lin and Johnson, 2015) and inhibit innovative behavior. In summary, we hypothesize the following:

H_4_: Team psychological safety cross-level moderates the relationship between workplace anxiety and employee innovation behavior. Thus, the relationship is stronger when the team's psychological safety is lower.

### 2.5. Moderated mediation effect

Combined with H_3_ and H_4_, we propose that team psychological safety moderates self-serving leadership through the influence of workplace anxiety on employee innovation behavior. Specifically, when team psychological safety is high, members are more likely to have positive interpersonal relationships (Banks et al., 2014). In other words, team members are less concerned about interpersonal risks, including negative impressions of themselves, arising from workplace anxiety caused by self-serving leadership. Thus, high levels of team psychological safety can effectively relieve employees' fear of a negative impact (Moake et al., 2019); self-control of resource consumption provides relief. In addition, as an organizational support resource, team psychological safety can help replenish the resource depletion caused by the anxiety of team members (Grandey et al., 2012). As a result, team members can put more resources into their work, stimulating innovative behaviors (Amabile, 1993).

In contrast, when the team lacks psychological safety, team members worry that revealing their negative emotions caused by the leader's self-serving behavior will lead to too many interpersonal risks. Therefore, they attempt to suppress their feelings and reinforce self-control. At the same time, the workplace anxiety of team members caused by the self-serving leadership forces employees to allocate their resources to the source of their stress, thereby reducing team members' focus on work (Eysenck et al., 2007). Moreover, this focus results in the reduction of resource input in follow-up work (Weiss and Cropanzano, 1996) because employees use simpler cognitive strategies and produce mediocre ideas (Sun et al., 2018), thus inhibiting the innovative behaviors of team members (Zhang et al., 2014). Therefore, we propose the following:

H_5_: Team psychological safety moderates the mediation of workplace anxiety on the relationship between self-serving leadership and employee innovation behavior. The mediating effect for teams with low psychological safety is stronger than for teams with high psychological safety.

**Figure 1:**
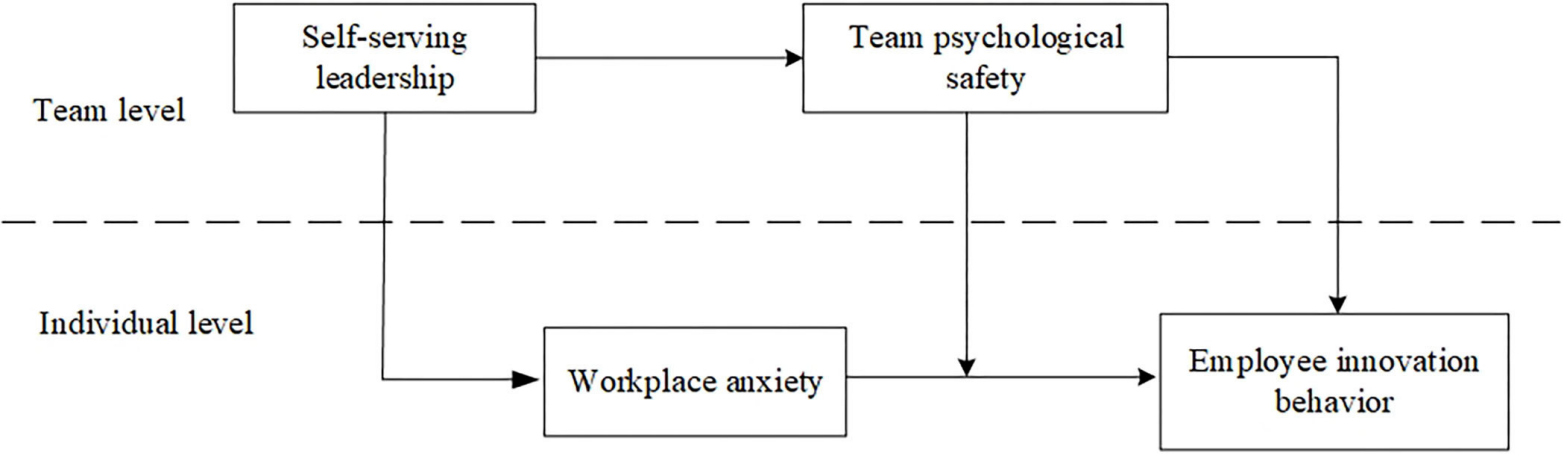
Theoretical model.

Figure 1 illustrates the hypothesized model.

## 3. Method

### 3.1. Procedure and samples

Data were collected from staff on active duty at different organizations in China *via* mail. To obtain as large a sample as possible, this study adopted the non-probability sampling method, combining convenience sampling and snowball sampling methods, to collect research data. Through social relations, researchers found participants who were willing to participate in our questionnaire survey, such as friends, classmates, and recently graduated students. We then asked them to be team contacts and explained to them the study's purpose, methods, and requirements, as well as tasked them with inviting their supervisors and colleagues to participate. The contacts chose their own time to distribute the questionnaires based on the situation of the enterprises, and they collected the questionnaires uniformly after they were completed.

The study was conducted in two main ways. First, paper-based questionnaires were distributed in batches, either in person or by mail. The questionnaires were delivered in sets, and the researchers placed the questionnaires for each team leader and their subordinates in a single envelope, which contained one questionnaire description, five questionnaires for team employees at time point T1, five questionnaires for team employees at time point T2, and one questionnaire for the team supervisor at time point T3. Team leader and employee questionnaires were marked T1, T2, and T3 on the envelope cover at different time points. Both team leader questionnaires and team employee questionnaires were reserved for coding matching. Second, electronic questionnaires were distributed. We informed the contact person of the filling requirements in advance, especially the time interval and questionnaire code, to ensure that the questionnaires at the three-time points could be matched and classified as the T1, T2, and T3 questionnaires. After filling it out, the contact person sent it to the researcher through the network.

To ensure the validity of the questionnaire and avoid the ideological burden of participants in the process of filling out the questionnaire, we briefly trained the contact individuals before the distribution of the questionnaire, ensuring the anonymity and academic nature of the survey. We also stressed the confidentiality and anonymity of the questionnaire to the respondents in the questionnaire filling instructions. At the same time, the double-sided tape was attached to each envelope, which was placed and sealed by the subjects themselves after filling out the questionnaire. Meanwhile, the subjects were told that there were no right or wrong answers. In addition, to improve the recovery rate of the questionnaire, we provided a reward of RMB ¥10 (about USD $1.43) to each participant.

The data of this research collection adopted three phases and the supervisor–subordinate pair to eliminate common method variance (Podsakoff et al., 2003, 2012). We considered an interval of 2 weeks between the three phases to be suitable (for a similar approach, see Eva et al., 2020). At Time 1 (T1), we requested subordinates to assess their perceptions of leadership and offer demographic information. We distributed 540 questionnaires to 108 teams, and 466 valid questionnaires were collected from 102 teams. At Time 2 (T2), which took place 2 weeks later, subordinates assessed workplace anxiety and team psychological safety. We distributed 466 questionnaires at T2 and received 418 questionnaires from 96 teams. Finally, at Time 3 (T3), which took place 2 weeks after Time 2, the supervisors evaluated the subordinates' innovation behaviors and the team's background information. A total of 418 questionnaires were distributed to 96 team leaders at T3, and the questionnaires of 88 team leaders and 405 employees were returned. After matching the three-wave questionnaires, we excluded the questionnaires that had teams with <3 members, answers with regularity, and vacancy as primary variables. The final sample used for this study consisted of 86 supervisors and 392 subordinates. The effective feedback rates of team leaders and employees were 89.58 and 72.59%, respectively. Each supervisor evaluated an average of 4.56 subordinates. Of the 392 employees, 58.2% were female, and 78.8% of participants had a bachelor's or junior college degree. The participants' ages were 25 years old or below (11%), 26–35 years old (68.4%), 36–45 years old (15.8%), and 46 years or older (4.8%). Regarding tenure, 56.4% were with the company for 5 years or less, 30.6% for 6–10 years, 8.7% for 11–20 years, and 4.3% for 21 years or more. In addition, participants reported working with their supervisor for 2 years or less (47.4%), 3–5 years (33.4%), 6–10 years (15.8%), and 11 years or more (3.3%). The average team size of the 86 teams in this study was 6.90.

### 3.2. Measures

Since Western countries created the measurements adopted in our research, we adopted the back-translation procedure (Brislin, 1970) to maintain consistency between the Chinese and English scales. Unless demographic variables were included, all items used a five-point Likert scale (1 = strongly disagree to 5 = strongly agree).

#### 3.2.1. Self-serving leadership

A four-item scale for measuring SL was developed by Camps et al. (2012). Sample items include “My superior does not show consideration for their followers, only for themselves.” Cronbach's α for this section in the present study was 0.93. Given that SL was a team-level construct, we adopted within-group reliability (ICC1), group mean reliability (ICC2), and within-group agreement indices (rwg) to evaluate the viability of aggregating the individual-level data on SL to the group-level. ICC1, ICC2, and rwg were 0.56, 0.85, and 0.81, respectively. The results exceeded the acceptable standards of 0.12, 0.47, and 0.70 (James, 1982), justifying the aggregation of SL.

#### 3.2.2. Workplace anxiety

A two-item scale for measuring WA was developed by Kouchaki and Desai (2015) and adopted to measure WA. Items are “I feel anxious at work” and “I feel nervous at work.” Cronbach's α for this section in the present study was 0.92.

#### 3.2.3. Team psychology safety

A seven-item scale for measuring TPS was developed by Edmondson (1999). Sample items include “Members of this team can bring up problems and tough issues.” Cronbach's α for this section in the present study was 0.90. ICC1, ICC2, and rwg of TPS were 0.50, 0.82, and 0.90, respectively, above the thresholds. The result justified the aggregation of TPS.

#### 3.2.4. Employee innovative behaviors

A six-item scale for measuring EIB was developed by Scott and Bruce (1994). Sample items include “At work, they search out new technologies, processes, techniques, and/or product ideas.” The Cronbach's α for this section in the present study was 0.94.

#### 3.2.5. Control variables

According to other research (Sun et al., 2018), age, gender, education, tenure, and time working with their current direct supervisors were controlled for in our analyses. In addition, because team size can affect the interaction of team members (Wheelan, 2009), team size was considered as a control variable.

## 4. Results

### 4.1. Confirmatory factor analysis

Before verifying hypotheses, we conducted confirmatory factor analyses (CFAs) using Amos 24 to assess the distinctness of these variables. The results presented in Table 2 indicate the proposed hypothesized measurement model that yielded an acceptable fit (χ^2^ = 324.20, df = 146, CFI = 0.97, TLI = 0.96, RMSEA = 0.06). This result showed that the distinctiveness of the four variables of the hypothesized model (self-serving leadership, workplace anxiety, psychological safety, and employee innovation behavior) was supported.

**Table 2:** Confirmatory factor analysis and model comparison.

**Model**	***χ^2^***	**df**	**CFI**	**TLI**	**RMSEA**
1.Four factors: SL; TPS; WA; EIB	324.20	146	0.97	0.96	0.06
2.Three factors: SL + TPS; WA; EIB	1,516.15	149	0.76	0.72	0.15
3.Three factors: SL; TPS; WA + EIB	800.07	149	0.89	0.87	0.11
4.Two factors: SL; TPS + WA + EIB	2,120.05	151	0.65	0.61	0.18
5.Two factors: SL + TPS + WA; EIB	2,009.45	151	0.67	0.63	0.18
6.One factors: SL + TPS + WA + EIB	3,294.38	152	0.45	0.38	0.23
Common factor	232.92	127	0.98	0.98	0.05

SL, self-serving leadership; TPS, team psychological safety; WA, workplace anxiety; EIB, employee innovative behavior.

### 4.2. Common method variance

To reduce the possibility of common method variance (CMV), we conducted time-lagged and multi-source experiments to collect data (Siemsen et al., 2009). We evaluated CMV using the Harman single factor test in SPSS 23.0, resulting in 39.09% in the first unrotated factor. Because the value was below 40%, we concluded that the research was effectively free of common method bias (Ashford and Tsui, 1991). Moreover, we introduced one common factor based on a four-factor model. If the new model's (i.e., adding the common factor) fit index improved significantly in contrast with the hypothesized model, then this indicates the existence of CMV. The results indicated that the fit indexes of the new model (CFI = 0.98, TLI = 0.98, and RMESA = 0.05) were not significantly better (both were <0.02) (Williams et al., 1989). Thus, the influence of CMV was not severe in this study.

### 4.3. Descriptive statistics and correlations

Table 3 lists the means, standard deviations, and correlations among the study variables. We found a negative relationship between WA and EIB (β = −0.31, *p* < 0.01) and SL and TPS (β = −0.52, *p* < 0.01).

**Table 3:** Means, standard deviations, and correlations.

	***M***	***SD***	**1**	**2**	**3**	**4**	**5**	**6**
**Individual-level**								
(1) Gender	0.42	0.49						
(2) Age	2.15	0.66	0.13^*^					
(3) Education	3.13	0.46	0.02	−0.16^**^				
(4) Tenure	1.61	0.82	0.05	0.62^**^	−0.16^**^			
(5) Work with leader	1.75	0.84	0.03	0.39^**^	−0.16^**^	0.53^**^		
(6) WA	2.69	1.18	−0.03	−0.05	0.05	−0.06	−0.03	
(7) EIB	3.71	0.86	0.06	0.03	0.03	−0.01	−0.01	−0.31^**^
**Team-level**								
(1) Team_size	2.10	0.53						
(2) SL	1.92	0.88	−0.06					
(3) TPS	3.70	0.63	0.16	−0.52^**^				

n = 392 individuals, N = 86 teams. For gender, male = 1, female = 0. For age, 25 years or younger = 1, 25–30 years = 2, 36–45 years = 3, 46 years or older = 4. For education, Junior high and below = 1, Senior High School (Vocational high School) = 2, Junior college and Undergraduate = 3, Graduate degree = 4. For employee tenure, ≤ 5 years = 1, 6–10 years = 2, 11–20 years = 3, ≥21 years = 4. For the times work with supervisor, ≤ 2 years = 1, 3–5 years = 2, 6–10 years = 3, ≥11 years = 4. For Team size, ≤ 4 people = 1, 5–9 people = 2, 10–14 people = 3, more than or equal to 15 people = 4. ^*^p < 0.05, ^**^p < 0.01. SL, self-serving leadership; TPS, team psychological safety; WA, workplace anxiety; EIB, employee innovative behavior.

### 4.4. Hypothesis testing

We used HLM 6.08 to test our hypotheses in this study. When examining the main effect and the mediating effect, both team-level and individual-level variables were processed using grand-mean-centered analysis, as recommended by Hofmann and Gavin (1998) and Enders and Tofighi (2007). When testing for interaction effects, team-level variables were processed by grand-mean centering, and individual-level variables were processed by group-mean centering. Table 4 provides the results of the regression. First, two null models were tested to confirm whether workplace anxiety and employee innovation behavior have significant variance across groups. The Null Model 1 results revealed that the between-group variance of workplace anxiety (τ00) was 0.41 and the within-group variance (σ^2^) was 0.99, $\chi_{\left(85\right)}^{2}$ = 245.81, and *p* < 0.001, manifesting that 29.29% of the variability in workplace anxiety can be attributed to the groups. Similarly, the Null model 2 results revealed that the between-group variance of employee innovation behavior (τ00) was 0.40 and the within-group variance (σ^2^) was 0.34, $\chi_{\left(85\right)}^{2}$ = 550.50, and *p* < 0.001, confirming that 54.05% of the variability in employee innovation behavior can be attributed to the groups.

**Table 4:** HLM results for hypothesis testing.

**Variable**	**WA**			**EIB**							

	**Null model 1**	**M1**	**M2**	**Null model 2**	**M3**	**M4**	**M5**	**M6**	**M7**	**M8**	**M9**
Intercept	2.69^***^	2.69^***^	2.69^***^	3.71^***^	3.71^***^	3.71^***^	3.71^***^	3.71^***^	3.71^***^	3.71^***^	3.71^***^
**Individual-level**											
Gender		−0.03	−0.05		0.02	0.04	0.02	0.01	0.02	0.01	−0.00
Age		−0.07	−0.06		0.07	0.07	0.06	0.07	0.07	0.07	0.06
Education		0.1	0.05		0.09	0.11	0.10	0.1	0.11	0.10	0.13
Tenure		0.00	−0.00		−0.04	−0.05	−0.04	−0.06	−0.06	−0.06	−0.03
Work with leader		0.02	0.01		0.01	0.02	0.02	0.05	0.04	0.05	0.04
WA							−0.10^**^		−0.08^*^	−0.09^*^	−0.06
**Team-level**											
Team-size		−0.24	−0.21		0.19	0.15	0.16	0.07	0.07	0.06	0.08
SL			0.42^***^			−0.35^***^			−0.16^*^		
TPS								0.60^***^	0.43^***^	0.55^***^	0.59^***^
**Cross-level interaction variable**											
WA*TPS											0.19^*^
**Variance decomposition**											
Within-group variance σ^2^	0.99	1.00	1.01	0.34	0.34	0.34	0.34	0.34	0.33	0.33	0.32
Between-group variance τ00	0.41	0.41	0.27	0.40	0.4	0.31	0.35	0.27	0.24	0.25	0.27
Chi-square	245.81	235.69	183.90	550.50	538.50	431.83	485.50	387.45	352.38	365.22	409.04
Deviance	1,201.45	1,213.47	1,197.76	843.64	860.13	845.72	856.55	836.23	832.77	831.61	830.01

The regression coefficients are all non-standardized coefficients under robust standard errors. ^*^p < 0.05, ^**^p < 0.01, ^***^p < 0.001. SL, self-serving leadership; TPS, team psychological safety; WA, workplace anxiety; EIB, employee innovative behavior. WA^*^TPS denotes the interaction item for WA and TPS.

Subsequently, we tested the first hypothesis. We included control variables and self-serving leadership in the null model 2. As indicated in Model 4, self-serving leadership was negatively correlated with EIB (γ = −0.35, *p* < 0.001). This finding supported H1.

We tested H_2_ and H_3_ following the study of Baron and Kenny (1986). First, the independent variable (SL) is correlated with the dependent variable (EIB), which was proved by H_1_. Second, there should be a significant relationship between the independent variable (SL) and the mediator (TPS), as SL and TPS are both team-level variables. A single-level regression analysis was conducted using SPSS 23.0, with SL as the independent variable, TPS as the dependent variable, and team size as the control variable. There was a relationship between SL and TPS (γ = −0.36, *p* < 0.001). Third, the mediator (TPS) is significant in predicting the dependent variable (EIB) (Model 6, γ = 0.60, *p* < 0.001); finally, after adding the mediator variables TPS and WA into the model, the impact of the independent variable on the dependent variable becomes significant or non-significant. As indicated in Model 7, the effect of SL on EIB decreased from −0.35 (see Model 4) to −0.16 but was still significant, indicating that TPS has a partially mediating effect between SL and EIB. Therefore, this finding supported H2.

Similarly, the same procedure was adopted to verify H3: First, SL was related to EIB, which was proved by H_1_; secondly, as shown in Model 2, there was a relationship between the independent variable (SL) and the mediator (WA) (γ = 0.42, *p* < 0.001). Third, the mediator (WA) is significant in predicting the dependent variable (EIB) (Model 5, γ = −0.10, *p* < 0.01). Finally, after adding the mediator variables WA and TPS into the model, the association of the independent variable with the dependent variable becomes significant or non-significant. As indicated in Model 7, the effect of SL on EIB decreased from −0.35 (see Model 4) to −0.16 but was still significant, indicating that WA has a partial mediates effect between SL and EIB. Therefore, H3 was supported.

We further tested the cross-layer indirect effect of team psychological safety and workplace anxiety by following the recommendations of Preacher and Selig (2012) and using bootstrap analysis. In addition, we used R 3.6.3, and Monte Carlo repeated sampling was set to 20,000. The results showed that the mediating effect of self-serving leadership on employee innovation behavior *via* team psychological safety was −0.17, with a 95% CI [−0.29, −0.07], which did not include 0. Thus, this finding supported H_2_. Similarly, the mediating impact of self-serving leadership on employee innovation behavior through workplace anxiety was −0.22, with a 95% CI [−0.44, −0.05] and did not include 0. This result supported H_3_.

**Figure 2:**
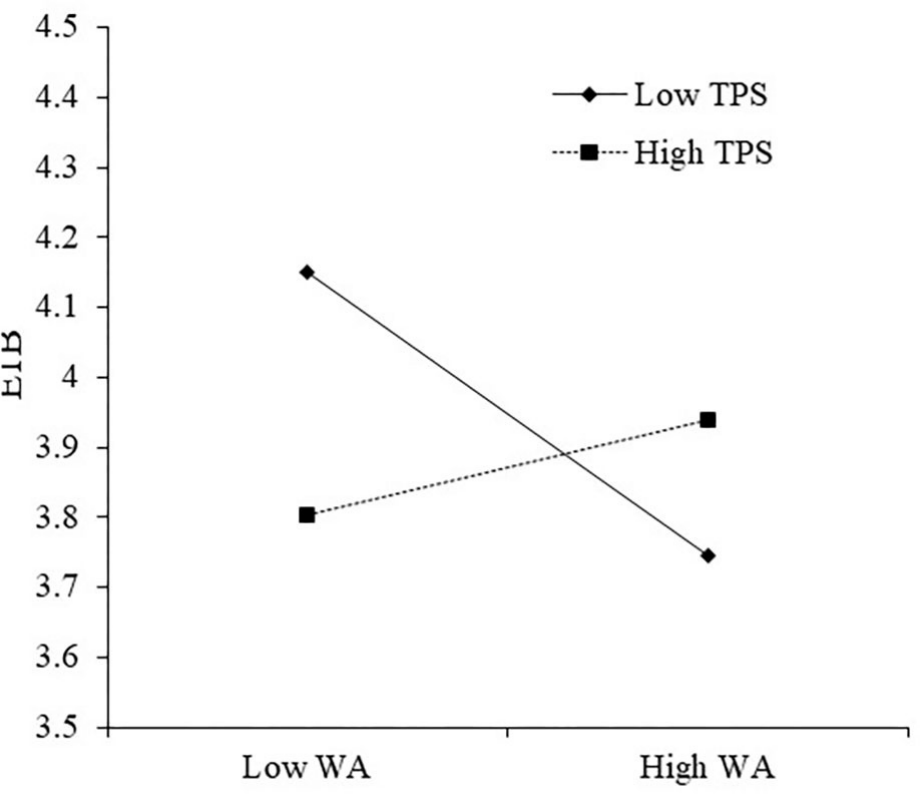
Moderating role of TPS on the relationship between workplace anxiety and employee innovative behaviors.

Next, we examined H_4_, which states that the impact of workplace anxiety on employee innovation behavior is moderated by team psychological safety. As presented for Model 9 in Table 4, the interaction between team psychological safety and workplace anxiety is significant to employee innovation behavior (γ = 0.19, *p* < 0.05). This finding indicated that the impact of workplace anxiety on employee innovation behavior is moderated by team psychological safety. We then used Aiken and West's (1991) recommendations to clarify the form of the interaction at two levels of team psychological safety. Figure 2 shows the impact of workplace anxiety on employee innovation behavior under M + SD and M – SD of team psychological safety. The interaction plot indicates a more robust relationship (simple slope = −0.17, *t* = −2.44) between workplace anxiety and employee innovation behavior when team psychological safety is low. However, when team psychological safety is high (simple slope = 0.06, *t* = 1.25), the influence of workplace anxiety on employee innovation behavior is not significant. However, the slope difference is significant (Δslope = 0.23, *p* < 0.05). Thus, H_4_ was supported.

Finally, we used R3.6.3 to test H5 and determine whether the mediated relation between self-serving leadership and EIB *via* workplace anxiety is moderated by psychological safety. Table 5 demonstrates that when team psychological safety is high, the mediating effect of workplace anxiety between self-serving leadership and EIB was 95% CI [−0.02, 0.06], including 0. However, when team psychological safety was low, the mediating effect of workplace anxiety was 95% CI [−0.11, −0.01], excluding 0. In addition, the between-group difference was 95% CI [0.01, 0.16], excluding zero. These results indicated that moderated mediation was supported. Therefore, these results supported H5.

**Table 5:** Moderated mediation testing.

**Dependent**	**TPS**	**Effect**	**SD**	**Low 95% CI**	**High 95% CI**
EIB	High	0.02	0.02	−0.02	0.06
	Low	−0.05	0.03	−0.11	−0.01
	Difference	0.07	0.04	0.01	0.16

SL, self-serving leadership; TPS, team psychological safety; WA, workplace anxiety; EIB, employee innovative behavior. WA^*^TPS denotes the interaction item for WA and TPS.

## 5. Discussion

Employee innovation behavior is key for organizational innovation, and how to motivate employee innovation behavior has garnered wide attention from numerous researchers and organizations. Leadership style plays a critical role in predicting employee innovation behavior (Zhang X., 2010); however, only a few studies explored the influence of self-serving leadership on employee innovation behavior. Therefore, some scholars recommend exploring the influence of self-serving leadership on employees' innovative behavior (Yang et al., 2020).

This study integrated the theories of social information processing, conservation of resources, and ego depletion in 392 employees from 86 teams as research samples to investigate mainly questions (Q1: How does self-serving leadership impact employee innovation behavior? Q2: What is the mediating mechanism in the impact of self-serving leadership on employees' innovative behavior? Q3: What are the boundary conditions for the relationship between self-serving leadership and employee innovation behavior?). To answer the above questions and respond to the advice of Li et al. (2021), this study constructed a cross-level model of two factors explaining the association of self-serving leadership with employee innovation from the perspectives of team psychological safety (team climate) and workplace anxiety (employee emotion).

The results showed that self-serving leadership has a significant detrimental influence on employee innovation behavior. As Liu et al. (2012) demonstrated, negative leadership influences employees' innovation negatively.

Next, based on social information processing theory (Salancik and Pfeffer, 1978), the social information formed by the leadership impacts the working environment and then affects the individual's attitude and behavior. From the perspective of social information processing, team psychological safety, as a kind of working environment, is an important intermediary mechanism between the impact of leadership on individual behaviors or attitudes (Zhang and Guo, 2021). According to this argument, we found the mediating role of team psychological safety in the relationship between self-serving leadership and employees' innovative behavior. As Yi et al. (2017) demonstrated, team psychological safety plays a mediating role between leadership and employee behavior.

Furthermore, psychologists believe that emotions play a critical role in predicting human behavior (Ashkanasy and Humphrey, 2011). In work scenarios, various work events trigger organizational members' emotional responses, which affect their work attitudes and behaviors (Weiss et al., 1999). Meanwhile, according to the conservation of resources theory, state anxiety consumes more cognitive resources than any other negative emotion (Ferris et al., 2008). It reduces employees' investment of cognitive resources in innovation and negatively affects their innovation behavior. The results confirm the mediating effect of workplace anxiety between self-serving leadership and employees' innovative behavior.

Finally, team psychological safety can apply to employees' perceptions of interpersonal risks and emotional expression (Grandey et al., 2012). Based on this argument and ego depletion theory, this research proposed that team psychological safety is a kind of social support and work resource at the team level (Halbesleben et al., 2014). Members can boldly express their emotions, effectively relieving the psychological pressure of expressing their anxiety and making their psychological state more stable, thus reducing the impact of negative emotions (Wei et al., 2019). Empirical results also support our hypothesis.

### 5.1. Theoretical contributions

The study has several theoretical contributions. First, this study constructed a conceptual model indicating that self-serving leadership impacts employee innovation behavior. It explored the trickle-down effect of self-serving leadership on employee innovative behavior from a new perspective. Employee innovative behavior as a kind of extra-role behavior (Wang and Chang, 2017), most studies pay attention to the effect of positive leadership on innovative behavior; however, there are not enough studies exploring the effect of negative leadership on employees' innovative behavior. Meanwhile, previous scholars who studied self-serving leadership mostly explored the effects of such leadership on subordinates' behaviors, such as counterproductive behaviors (Mao et al., 2019b), deviant behaviors (Zhou et al., 2021), and organizational citizenship behaviors toward leaders (Ritzenhöfer et al., 2019). Those research neglected the effects of self-serving leadership on employee innovative behaviors. This study emphasizes the antecedents of innovative employee behavior. The findings further revealed that the more self-interested the leader, the greater the likelihood of inhibiting innovative employee behavior. Specifically, this study points out that the self-serving behavior of leaders will elicit employees' insecurity and the perception of being underappreciated. Therefore, employees are less willing to take responsibility and focus on their work, which will prompt employees' risk-averse behavior and thus inhibit their innovative behavior. Accordingly, the influence process of self-serving leadership on employees' innovative behavior is revealed, and the role of negative leadership on individual innovative behavior is explored in depth in organizational situations, which is a useful addition to previous studies.

Second, previous studies showed that team-level leaders can impact employee innovation behavior through individual and team paths (Li et al., 2021). This study tests the cross-level indirect effects of two factors on the association of self-serving leadership with employee innovation based on the perspectives of team psychological safety (team climate) and workplace anxiety (employee emotion). Based on social information processing theory, the work environment of team members is an important information source that affects the effectiveness of their behaviors (Salancik and Pfeffer, 1978). Team members shape their shared perceptions of team atmosphere through social information clues such as how leaders distribute team benefits, thus becoming information sources affecting the effectiveness of their subsequent behaviors. In addition, according to the conservation of resources theory, since leaders meet their needs by exploiting employee interests, they can be seen as a threat to employees. This situation creates a stress response in subordinate employees, leading to an adverse psychological state. As a result, members spend a considerable amount of time and energy dealing with negative emotions, reducing their energy and resources to devote to innovation. The results of this study help unearth the “black box” of the association between self-serving leadership and employee innovation behavior and effectively explain the underlying mechanisms of this relationship.

Finally, psychological safety can affect team members' shared perceptions of their environment. As an organizational support resource, team psychological safety is beneficial to reducing members' perceptions of suffering from negative interpersonal interactions during emotional expression and provides employees with an opportunity to recover their psychological resources. Team members can make up for resource loss caused by anxiety (Grandey et al., 2012) so that employees have more resources to invest in the follow-up work, thus influencing their attitudes and behaviors. Therefore, this study applied ego-depletion theory to team psychological safety (a moderating variable in the theoretical construction that plays a positive role in improving team communication and cooperation) and analyzed the moderating role played in the link between workplace anxiety and employee innovation behavior. The results showed that team psychological safety negatively moderates the effect of workplace anxiety on employee innovation behavior. Specifically, a team lacking psychological safety can aggravate the loss of self-control resources caused by workplace anxiety. In addition, it can deplete employee resources for investing in follow-up work, thus exacerbating the adverse impact of workplace anxiety on employee innovation behavior. This finding helps clarify workplace anxiety's boundary condition affecting employee innovation behavior. Further, to some extent, it enriches the theory of team psychological safety research and leadership. It demonstrates that research situations involving team and individual interactions affect employee behavior and work results, improving the relationship between the factors' characteristics.

### 5.2. Managerial contributions

This study also has some implications for enterprise management practices. We found that self-serving leadership negatively impacts subordinates' innovation behavior and has a particular warning effect on leaders' daily management. If the team leader cannot effectively restrain their self-serving behaviors, employees will experience workplace anxiety. At the same time, self-serving leadership will adversely impact the team's psychological safety environment, reducing employees' innovative behaviors at work. Therefore, we recommend the following.

First, the organization's top managers must recognize the negative impact of leaders' self-serving behaviors and actively take measures to curb them. Moreover, organizations should emphasize the ethical character of leadership in team leader selection and avoid appointing individuals with self-serving behavior tendencies as team leaders. The study indicated that the power of leadership is directly proportional to selfish behavior (Bendahan et al., 2015). Thus, top managers should take measures to minimize the adverse influence of power, improve the constraint and oversight systems for exercising power, and prevent power from being abused to advance vested interests.

Second, this study confirmed that workplace anxiety has an important mediating effect on the link between self-serving leadership and employee innovation behavior, which provides insights for organizations so that they can mitigate the harmful effects of self-serving leadership. Therefore, it is necessary to strengthen the attention and management of employees' workplace anxiety, implement employee care plans (such as providing psychological counseling services, increasing micro-breaks at work, and so on), and promote the recovery of employees' psychological resources. Furthermore, a positive and open corporate culture and atmosphere can improve the psychological state of employees more effectively (Cheng and McCarthy, 2018). Therefore, organizations should abandon the concept of “profit first and opportunistic,” initiate corporate social responsibility, and build a positive corporate culture.

Finally, team psychological safety serves as a partial mediator in the association of self-serving leadership with employee innovation behavior. The results showed that improving a team's psychological safety and promoting subordinates' innovation behaviors are feasible. The organization could build an inclusive corporate environment. Studies showed that leadership inclusiveness promotes team psychological safety (Hirak et al., 2012). Therefore, managers should accept criticism with an open mind, tolerate the different opinions of subordinates, and make employees believe that leaders will not retaliate or take personal revenge. Moreover, organizations should develop a system to encourage employees to make bold suggestions so that employees can muster up courage to take “interpersonal risks.” In addition, they should encourage all team members to dare to speak their minds and give timely praise and affirmation to those who provide constructive feedback, thus improving team psychological safety.

### 5.3. Limitations of this study and future research direction

Our research has several limitations. First, we based this research on the Chinese context; in different cultural contexts, employees' understanding of self-serving leadership may be different (Yang et al., 2020), which limits the generalizability of our research conclusions. Therefore, future research should consider using samples from Western cultural contexts to test this study's reliability. Second, the measurement of self-serving behavior is sensitive. Therefore, self or subordinate reporting causes deviation. Therefore, future studies could use different methods (such as in-depth interviews and other qualitative research methods) to collect data on self-serving leadership, further exploring the mechanism and boundary conditions under which self-serving leadership is related to employee innovation behavior. Third, this study mainly focused on the individual and team levels and did not consider the influence of organizational variables on the research model. Future research could incorporate organizational variables such as corporate culture and organizational ethical climate into the research model for careful consideration of the systematic impact of each factor on innovation behavior. Fourth, although this study adopted two independent sources (direct leaders of the work team and team members) and two-time points to obtain research data, we can eliminate the potential impact of common method bias on research results to some extent (Podsakoff et al., 2003). However, in essence, this research was a cross-sectional study. Thus, we cannot make causal inferences from the results of this study. Although top-down processes are more likely to occur in a work team, team members also influence leaders, which is a bottom–up process (Kozlowski and Klein, 2000). Future research could adopt an experimental or longitudinal study design to explore the relationship between variables in this study. Finally, for the measurement of workplace anxiety, we chose a two-item scale developed by Kouchaki and Desai (2015), which has the advantage of being concise. Some other studies on workplace anxiety usually used the eight-item scale provided by McCarthy et al. (2016), which provides a more comprehensive assessment of employees' anxiety in the workplace by including dimensions of job performance, job competency, job outcome, and performance evaluation. In future studies, we will use other well-established scales to measure workplace anxiety more precisely to further ensure the accuracy of the study results.

## Data availability statement

The original contributions presented in the study are included in the article/supplementary material, further inquiries can be directed to the corresponding author.

## Author contributions

LL and ZW: conceptualization and funding acquisition. LW and ZW: data collection. ZW: formal analysis and writing–original draft preparation. LL and LW: writing–review and editing. All authors contributed to the article and approved the submitted version.
